# Delayed-Type Hypersensitivity Underlying Casein Allergy Is Suppressed by Extracellular Vesicles Carrying miRNA-150

**DOI:** 10.3390/nu11040907

**Published:** 2019-04-23

**Authors:** Magdalena Wąsik, Katarzyna Nazimek, Bernadeta Nowak, Philip W. Askenase, Krzysztof Bryniarski

**Affiliations:** 1 Department of Immunology, Jagiellonian University Medical College, 31-121 Krakow, Poland; magdalena.wasik@interia.pl (M.W.); katarzyna.nazimek@uj.edu.pl (K.N.); bernadeta.nowak@uj.edu.pl (B.N.); 2 Section of Rheumatology, Allergy and Clinical Immunology, Yale University School of Medicine, New Haven, CT 06520, USA; philip.askenase@yale.edu

**Keywords:** cow’s milk allergy, casein, cell-mediated reactions, delayed-type hypersensitivity, extracellular vesicles, miRNA-150

## Abstract

In patients with non-IgE-mediated milk allergy, a cellular mechanism of delayed-type hypersensitivity (DTH) is considered. Recent findings prove that cell-mediated reactions can be antigen-specifically inhibited by extracellular vesicles (EVs) carrying miRNA-150. We sought to establish a new mouse model of DTH to casein and test the possibility of antigen-specific suppression of the inflammatory reaction. To produce soluble antigenic peptides, casein was subjected to alkaline hydrolysis. DTH reaction to casein was induced in CBA, C57BL/6, and BALB/c mice by intradermal (id) injection of the antigen. Cells collected from spleens and lymph nodes were positively or negatively selected and transferred to naive recipients intravenously (iv). CBA mice were tolerized by iv injection of mouse erythrocytes conjugated with casein antigen and following id immunization with the same antigen. Suppressive EVs were harvested from cell cultures and serum of tolerized donors by means of ultrafiltration and ultracentrifugation for further therapeutic utilization. The newly established mouse model of DTH to casein was mediated by CD4+ Th1 cells and macrophages, while EVs produced by casein-tolerized animals effectively suppressed effector cell response, in an miRNA-150-dependent manner. Altogether, our observations contribute to the current understanding of non-IgE-mediated allergy to casein and of the possibilities to downregulate this reaction.

## 1. Introduction

Cow’s milk allergy is one of the most common food allergies in children. It is hard to precisely estimate the prevalence of milk allergy, because its symptoms are often mistaken with lactose intolerance, a non-immunological reaction [1]. Allergy to cow’s milk usually manifests itself as a type I hypersensitivity reaction that is IgE-mediated and occurs immediately after contact with the allergen in sensitized individuals. There is also a group of patients with onset delayed more than 20 h after the exposure to the allergen and where the reaction does not depend on IgE level [2,3,4]. This suggests involvement of another mechanism likely cell-mediated. 

Delayed-type hypersensitivity (DTH) to common allergens is far more difficult to diagnose than type I allergy and thus remains a challenge for clinicians. Symptoms of non-IgE-mediated allergy to food proteins are mostly gastro-intestinal and include malabsorption, bloody diarrhea, emesis, pallor, lethargy, and weight loss [1,5]. 

DTH, classified by Gell and Coombs as a type IV allergic reaction, has been widely studied in drug-induced models, and a new subclassification has been established according to the manifestation of the allergy and the type of cells involved in the reaction. Subtype IVa is mediated by Th1 lymphocytes and macrophages (manifested by bullous exanthem), while IVb by Th2 lymphocytes and eosinophils is accompanied by maculopapular exanthem. Subtype IVc is mediated by cytotoxic T lymphocytes, and either maculopapular or bullous exanthem can be observed as the clinical symptoms. Pustular exanthem is a hallmark of the IVd subtype, to which reaction depends on T lymphocytes producing IL-8 and GM-CSF that activate neutrophils [6]. However, it still remains an open question if DTH subtypes underlie the non-IgE-mediated allergy to casein.

Cow’s milk consists of around 30 different proteins, from which caseins constitute approximately 80% [7]. Currently, the most effective treatment of non-IgE-dependent allergy to cow’s milk is the elimination of the offending food and all products that may contain its proteins from child’s diet. If an infant with a cow’s milk allergy is breastfed, then it should also be eliminated from the mother’s diet. This method is a great burden for parents, since milk proteins, such as casein, can be found not only in dairy products but also in many processed foods [1,8]. Total avoidance of specific food may even predispose infants to develop an allergy by disturbing IgA-mediated oral tolerance [9,10]. 

Recently, it was proved that cell-mediated allergic reactions to haptens can be inhibited by exosome-like extracellular vesicles (EVs) carrying miRNA-150 [11]. Exosomes representing a population of small EVs have recently become the subject of increasing interest as a new means of intercellular communication. They are produced intracellularly in multivesicular bodies and are released by exocytosis. EVs can contain a “cargo” of miRNA that may be delivered to the acceptor cell and affect its physiology [12]. In a murine model of contact sensitivity to haptens, EVs carrying miRNA-150 had the capacity to antigen-specifically target effector T cells and suppress the inflammation [11]. These EVs were produced by a distinct population of CD8+ suppressor T (Ts) lymphocytes, but not from FoxP3+ regulatory T cells, after intravenous administration of syngeneic erythrocytes conjugated with hapten to naive mice. Moreover, they became antigen-specific when coated with IgM light chains, produced by B1 lymphocytes after contact immunization with the same hapten. The suppressive Ts cell-derived miRNA-150-carrying EVs and B1 cell-produced antibody light chains were described to act together as soluble T suppressor factor (TsF) [13]. Our preliminary data suggest that analogous tolerance mechanism mediated by Ts cell EV-contained miRNA-150 could also be induced in DTH to ovalbumin.

Our study aimed to describe the effector mechanisms and to standardize a new, mouse model of DTH to casein antigen. For this purpose, we established a method of obtaining a soluble casein (Cas) antigen that appears to be safe to use in an animal model of active immunization and to induce cellular inflammatory response. The next aim of our research was to test the possibility of antigen-specific inhibition of DTH reactions to protein antigen, such as Cas, in adoptive transfer and in active immunization. 

## 2. Materials and Methods

### 2.1. Mice

Mice of CBA, BALB/c, and C57BL/6 inbred strains were obtained from the breeding unit of the Faculty of Medicine, Jagiellonian University Medical College in Krakow, Poland. Animals were fed with casein-free chow supplied by Labofeed H (Kcynia, Poland). All experiments were conducted according to the guidelines of the Animal Care and Use Committee of the Jagiellonian University Medical College and Yale School of Medicine, New Haven, CT (Permit Number 07381) and under ethical approval of the 1st Local Ethics Committee in Krakow (approval no. 243/2015). Control and treatment groups consisted of 4–5 animals.

### 2.2. Casein Alkaline Hydrolysis

Insoluble bovine casein (Sigma-Aldrich, St Louis, MO, USA), containing all casein fractions, i.e., α-s1, α-s2, β, and κ-casein, was suspended in NaHCO_3_ (0.5 M) in a 1:8 ratio (w/v) and hydrolyzed in 37 °C for 5–7 h with occasional mixing. The solution was then kept at room temperature for subsequent 48 h. Next, all probes were centrifuged (3300 g, 15 min), and the soluble fraction was dialyzed to DPBS. The remaining pellet was resuspended in NaOH (1 M), adjusted to pH = 10 with HCl solution, mixed, and kept at room temperature for the next 24 h. Again, the resulting soluble fraction was processed as above. Apart from immunogenicity testing, soluble Cas fractions after hydrolysis with NaHCO_3_ or NaOH were mixed and then dialyzed to either DPBS (for coupling with erythrocytes) or 0.9% NaCl (for immunization). Yielded soluble Cas antigen was filtrated, and the protein concentration was then assessed with a UV spectrophotometer at 280 nm.

### 2.3. Active Immunization

Mice under light anesthesia were immunized with the Cas antigen by intradermal administration of a total of 200 μL of soluble Cas peptides (0.5 mg/ml) administered into 4 separate sides of the abdomen on Days 0 and 1. On Day 5, mice were challenged on both ears by intradermal administration of 10 μL of the same Cas solution. The increase in ear thickness was measured by a blinded observer using an engineer’s micrometer (Mitutoyo, Kawasaki, Japan) from 24 to 120 h later [14]. Background earlobe thickness was subtracted to obtain a value of ear thickness increase for each mouse, while nonspecific increase in earlobe thickness in non-immunized, but challenged littermates was subtracted from experimental groups to yield a net swelling value expressed as delta ± standard error (SE) [U×10^−2^ mm]. 

### 2.4. Adoptive Transfer of DTH Effector Cells

Cas-immunized mice (see above) were sacrificed on Day 5 by means of cervical dislocation under deep anesthesia. Effector cells were obtained from spleens and lymph nodes, and their single cell suspensions in DPBS were transferred intravenously to naive recipients (7 × 10^7^ cells per mouse). The next day, animals were ear-challenged by intradermal administration of 10 μL of soluble Cas, and subsequent earlobe thickness was measured as above [14].

### 2.5. Negative Selection Assay

DTH effector cell suspensions from spleens and lymph nodes of Cas-immunized mice were incubated with monoclonal antibodies specific for CD4 (rat IgG2b antibody, GK1.5 clone) or CD8 (rat IgG2b antibody, TIB-105 clone, both cell lines were cultured and produced monoclonal antibodies were chromatographically purified at Department of Immunology, Jagiellonian University Medical College, Krakow, Poland) markers. The rabbit complement (BIOMED, Lublin, Poland) was then added and suspensions were incubated for 1 h in a 37 °C water-bath. DTH effector cells were depleted of macrophages by triple filtration through a 70 μm nylon mesh. Selection was followed by Ficoll centrifugation to remove dead cells. Selected viable cells were suspended in DPBS and administered to naive mice intravenously in adoptive transfer (see above) [15].

### 2.6. Positive Selection Assay

Cell suspensions from spleens and lymph nodes of mice immunized or tolerized with Cas antigen or Cas-coupled erythrocytes, respectively, were positively selected on MiniMACS MS columns with microbeads coated with monoclonal antibodies anti-CD4 or anti-CD8 according to manufacturer’s procedures (Miltenyi Biotec, Bergisch Gladbach, Germany). Selected viable cells were suspended in DPBS and intravenously administered to naive mice in adoptive transfer (see above).

### 2.7. Induction and Separation of Ts cell EVs from Cell Supernatants

Mice, on Days 0 and 4, were injected intravenously with 10% suspension of syngeneic erythrocytes conjugated with Cas antigen in the presence of 1-ethyl-3-(3-dimethylaminopropyl) carbodiimide (EDC) for activation of coupling process. This induced a generation of miRNA-150-carrying EVs by Ts cells. On Days 7 and 8, Cas antigen without an adjuvant was administered intradermally into 4 separate sites of the abdomen (100 μg per mouse, see above) in order to induce production of specific IgM light chains by B1 lymphocytes. Spleens and lymph nodes of tolerized mice were harvested on Day 10 and their single cell suspensions, containing Ts cells and B1 lymphocytes, were cultured for 48 h in protein-free Mishell-Dutton medium (MDM, 2 × 10^7^ cells per ml, 37 °C, 5% CO_2_). Supernatants from cell cultures were then centrifuged twice (300× *g* and 3000× *g*, for 15 min) and filtered through 0.45 and 0.22 μm syringe filters (Merck Millipore, Burlington, MA, USA). Finally, EVs were concentrated by double ultracentrifugation in a Beckman L870M ultracentrifuge (100,000× *g*, 70 min, 4 °C) and resuspended in DPBS [15,16] for further experimental usage as Ts cell EVs. In some cases, EVs were incubated with anti-miR-150 (miRIDIAN Hairpin Inhibitor of Mouse mmu-miR150, Dharmacon, GE Healthcare, Lafayette, CO, USA) at 37 °C for 1 h, in a dose of 3 μg per eventual recipient mouse, to block the biological activity of miRNA-150, and then washed to remove excessive anti-miR-150 molecules [15]. Where indicated, cells of spleens and lymph nodes of tolerized mice were subjected to a positive selection assay, as described above. 

### 2.8. Induction and Separation of B1 Cell-Produced EVs 

Mice were injected intradermally on Days 0 and 1 with Cas antigen without adjuvant into 4 separate sites of the abdomen (100 μg per mouse, see above) in order to induce production of B1 cell EVs, coated with specific IgM light chains, but devoid of miRNA-150 [11]. Spleens and lymph nodes of immunized mice were harvested on Day 3 and single cell suspensions were cultured for 48 h in protein-free MDM (2 × 10^7^ cells per ml, 37 °C, 5% CO_2_). Supernatants from cell cultures were then centrifuged twice (300× *g* and 3000× *g*, for 15 min) and filtered through 0.45 and 0.22 μm syringe filters. B1 cell-produced EVs were concentrated by ultracentrifugation in a Beckman L870M ultracentrifuge (100,000× *g*, 70 min, 4 °C) and resuspended in DPBS. In some cases, B1 cell EVs were incubated overnight on ice with miRNA-150 (miRIDIAN Mimic Mouse mmu-miR-150, Dharmacon, GE Healthcare, Lafayette, CO, USA), in a dose of 3 μg per eventual mouse recipient, and then washed to remove excessive miRNA-150 molecules. 

### 2.9. Antigen-Affinity Chromatographic Separation of Cas-Specific EVs 

Soluble Cas antigen or purified anti-CD9 monoclonal antibodies (BD Biosciences, San Diego, CA, USA) were linked to cyanogen bromide-activated Sepharose 4FF (fast flow; Pharmacia, Uppsala, Sweden) according to the manufacturer’s procedure. Supernatant from tolerized mouse lymph node and spleen suppressive cell culture was then applied onto either Cas- or anti-CD9- Sepharose-filled columns. The fraction of nanovesicles that first passed through the column was collected as flow through (FT, i.e., Cas-non-binding or CD9 negative EVs), and, after column washing with DPBS, the fraction of nanovesicles that was eluted with 5 M guanidine HCl (pH = 4.7) was collected as eluate (i.e., Cas-binding or CD9 positive EVs). Both fractions were filtered through 0.45 and 0.22 μm filters and ultracentrifuged twice in DPBS (100,000× *g*, 70 min, 4 °C) [11].

### 2.10. Suppression of Effector Cells Tested in Adoptive Transfer

DTH effector cells collected as above from spleens and lymph nodes of Cas-immunized mice were incubated with Ts cell EVs for 30 min in 37 °C, which was followed by washing in DPBS and 70 μm nylon mesh filtration. Cell suspensions in DPBS were transferred intravenously to naive, anesthetized recipients in a ratio of 7 × 10^7^ cells per mice. The next day, animals were ear-challenged by intradermal administration of 10 μL of Cas and ear thickness was measured after 24 h, as described above.

Approximately 1.3 × 10^10^ EVs were used either in adoptive transfer per 7 × 10^7^ effector cells per mouse or as a single dose administered to actively immunized mouse. The exact quantity of EVs was estimated using nanoparticle tracking analysis as described in detail previously [11]. 

### 2.11. Cytometric Analysis of EVs and Antibody Light Chains

Aldehyde/sulfate latex beads of 4 µm size (Life Technologies, Thermo-Fisher Scientific, Carlsbad, CA, USA) were incubated in DPBS with Ts cell EVs in a total volume of 1 mL of DPBS for 2 h at room temperature with gentle agitation. Afterwards, EV-coated beads were blocked with 100 mM glycine for 30 min at room temperature with gentle agitation. After washing and resuspending in DPBS, EV-coated beads were stained with fluorescein isothiocyanate (FITC)-conjugated monoclonal antibodies against mouse kappa light chains (BD Biosciences, San Diego, CA) and/or phycoerythrin (PE)-conjugated monoclonal antibodies against mouse CD9, CD63, or CD81 tetraspanins (BD Biosciences, San Diego, CA, USA) and acquired by a BD FACSCalibur (BD Bioscience, San Jose, CA, USA).

### 2.12. Test of Different Routes of Therapeutic EV Administration and Active Tolerance Induction

Ts cell-derived EVs or B1 cell-produced EVs were administered to the Cas-immunized animals intradermally, intraperitoneally, intravenously or per os in equal doses (see above) at the peak of the allergic response, i.e., 24 h post-challenge. Ear thickness was measured up to 120 h after challenge by a blinded observer, unaware of the experimental protocol, using an engineer’s micrometer (Mitutoyo, Kawasaki, Japan). Otherwise, mice, 5 days prior to active immunization, had been injected intravenously with 10% suspension of syngeneic erythrocytes conjugated with Cas antigen, and subsequent ear swelling was elicited as above 5 days after immunization. 

### 2.13. MHC Criss-Cross Testing

DTH effector cells collected from Cas-immunized CBA, BALB/c, or C57BL/6 mice were incubated with Ts cell EVs obtained from CBA mice tolerized to Cas antigen. Next, EV-pulsed or non-pulsed effector cells were intravenously transferred to naive mice of respective strain. On the following day, mice were intradermally ear-challenged with Cas antigen, and ear thickness was measured as described above.

### 2.14. Statistical Analysis

Readings were done twice on both ears, and the inflammatory response induced in each mouse was expressed as a mean of the 4 measurements. After meeting of test assumptions, one-way analysis of variance (ANOVA) with post-hoc RIR Tukey test was used to evaluate the statistical significance between the groups with *p* < 0.05 taken as a minimum level of significance, which was marked in the figures as * *p* < 0.05; ** *p* < 0.01; *** *p* < 0.001; **** *p* < 0.0001.

## 3. Results

### 3.1. Soluble Casein Antigen Induces Allergic Reaction

**Figure 1:**
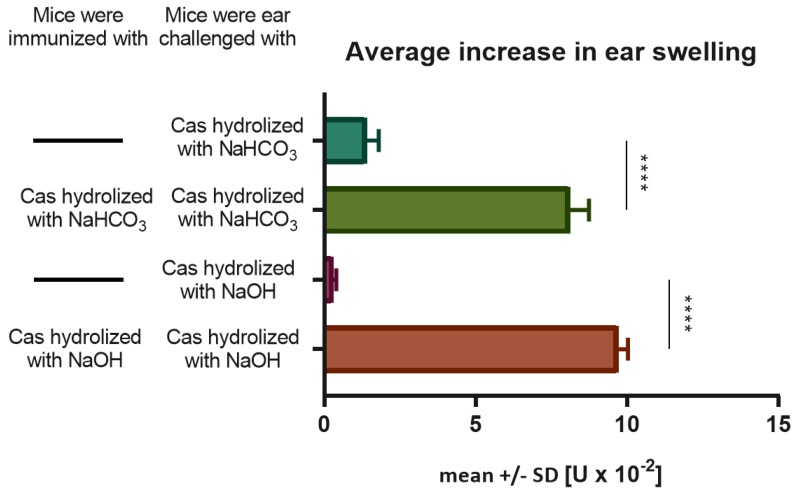
Immunogenicity of soluble casein (Cas) antigen obtained by alkaline hydrolysis with either NaHCO_3_ or NaOH. Mice had been intradermally (id) immunized with a saline solution of soluble Cas antigen (100 µg per mouse) 5 days before challenging by id administration of the same Cas solution (5 µg per earlobe). Twenty-four hours later ear swelling response was measured and expressed as mean ± SD [units (U) × 10^−2^ mm] (n = 4, N = 3). **** *p* < 0.0001.

Mice intradermally immunized and challenged with soluble Cas antigen fractions hydrolyzed in either NaHCO_3_ or NaOH, developed a significantly greater ear swelling response, when compared to the negative control group of mice only challenged with respective Cas fraction (Figure 1). In addition, ear swelling response peaked 24 h after challenge in immunized animals, which resembles DTH reaction. Thus, we assumed that Cas antigen preserves its immunogenicity during alkaline hydrolysis and is able to induce DTH reaction in mice after intradermal administration without an adjuvant. Besides, inflammatory reactions caused by immunization with both fractions of soluble Cas antigen were comparable, so we decided to mix them in order to use in further experiments.

### 3.2. CD4+ T Cells and Macrophages Mediate the Effector Phase of DTH Reaction to Cas Antigen

**Figure 2:**
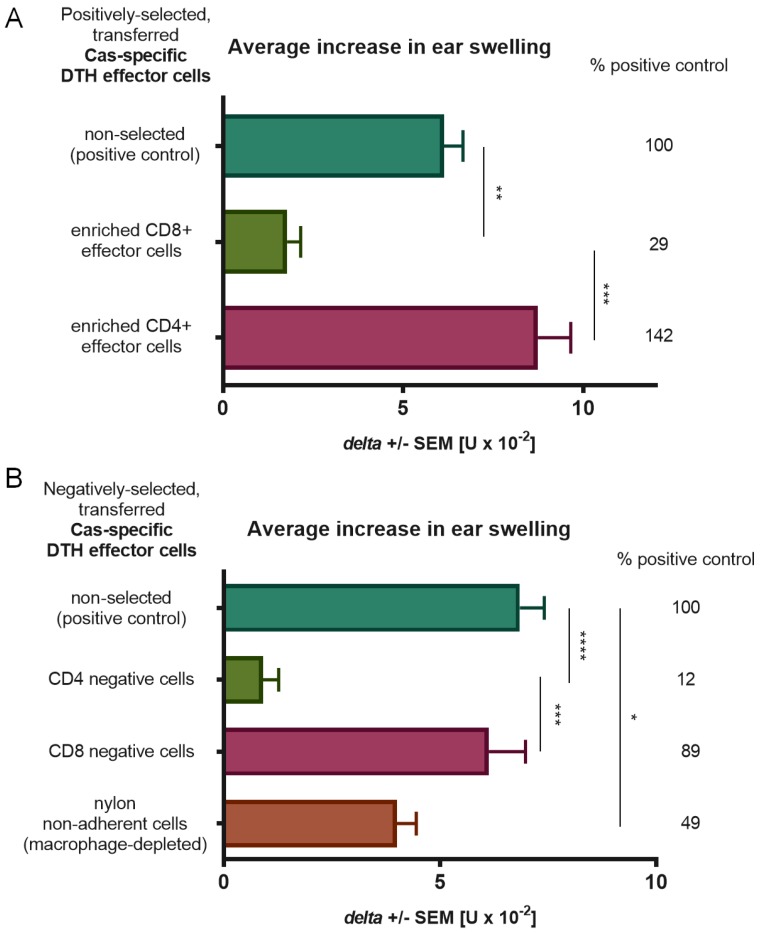
Phenotyping of effector cells of delayed-type hypersensitivity (DTH) to soluble casein (Cas) antigen. Mice had been intradermally (id) immunized with a saline solution of soluble Cas antigen (100 µg per mouse) 5 days before harvest of lymph nodes and spleens containing effector cells, which were then subjected to positive (**A**) or negative (**B**) selection assays by, respectively, magnetic-advanced cell sorting or depletion with either monoclonal antibodies and complement or nylon wool separation. Afterwards, selected effector cells were transferred to naive recipients, which 24 h later were challenged by id administration of the same Cas solution (5 µg per earlobe). After 24 h, ear swelling response was measured and expressed as delta ± SEM [units (U) × 10^−2^ mm] (n = 5, N = 2), * *p* < 0.05; ** *p* < 0.01; *** *p* < 0.001; **** *p* < 0.0001.

Phenotype of DTH effector cells mediating allergic reaction in Cas-immunized mice was assessed using negative and positive selection assays (Figure 2). Statistically significant decrease in ear swelling in comparison to a positive control was observed in mice depleted of CD4+ T lymphocytes or macrophages prior to adoptive transfer, which indicates that both cell populations are important for induction of DTH reaction to Cas antigen. The inflammatory response in mouse recipients of macrophage-depleted DTH effector cells was noticeably higher than that in groups deprived of CD4+ T cells, which is possibly caused by the activity of a recipient’s macrophages, which can still elicit inflammatory response of transferred effector CD4+ T cells.

### 3.3. CD8+ T Cells Are Responsible for Production of Suppressive EVs that Express CD9 and CD81 Tetraspanins and Are Specific to Casein due to Expression of Antibody Light Chains

**Figure 3:**
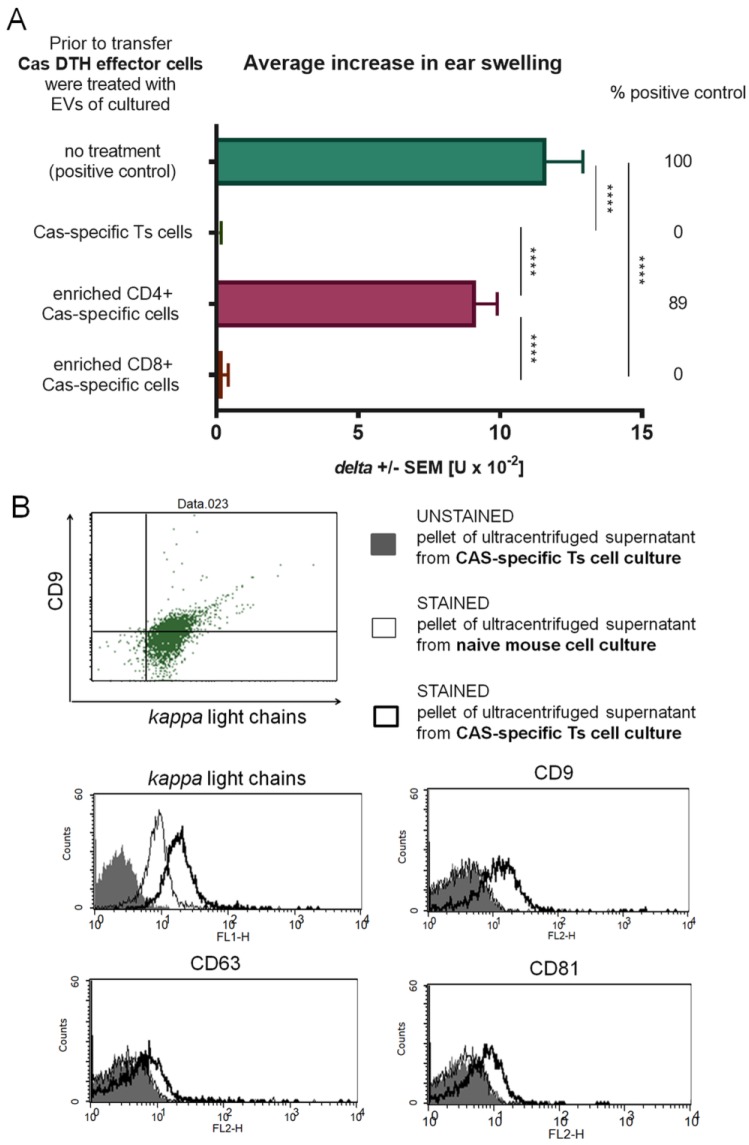

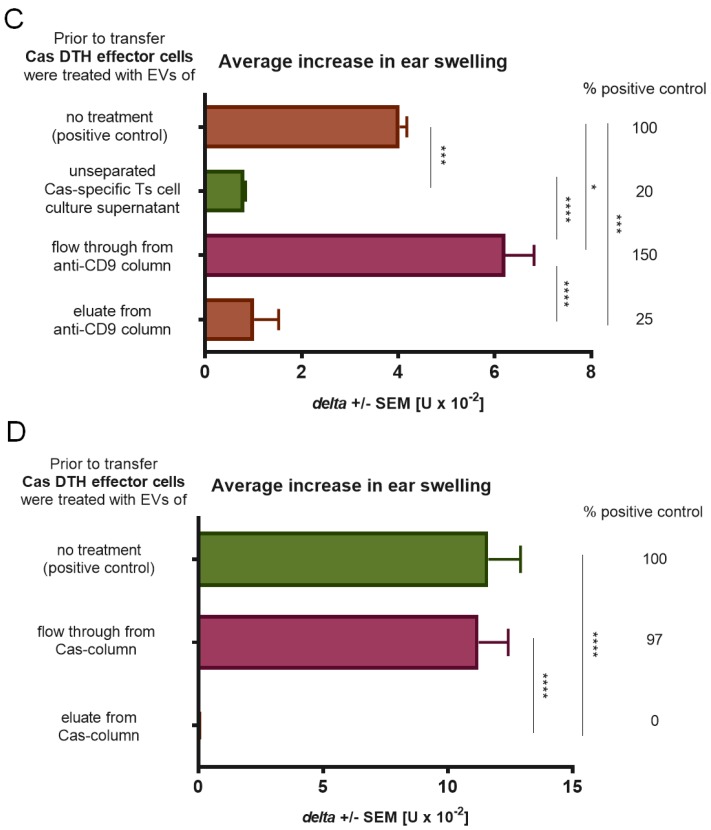
Suppression of delayed-type hypersensitivity (DTH) to soluble casein (Cas) antigen is mediated by CD8+ suppressor T (Ts) cell-derived extracellular vesicles (EVs) expressing CD9 and CD81 tetraspanins and Cas-specific antibody light chains. (**A**) Mice had been intravenously administered with Cas-coupled syngeneic red blood cells, followed by intradermal (id) immunization with a saline solution of soluble Cas antigen (100 µg per mouse) 3 days before harvest of lymph nodes and spleens containing Ts cells, which were then subjected to positive selection assay, i.e., magnetic-advanced cell sorting, and cultured for subsequent 48 h. The resulting supernatant was filtered and ultracentrifuged to concentrate Ts cell EVs, used to treat DTH effector cells prior to adoptive transfer to recipients, challenged 24 h later by id administration of the same Cas solution (5 µg per earlobe). After 24 h, ear swelling response was measured and expressed as delta ± SEM [units (U) × 10^−2^ mm] (n = 5, N = 2). (**B**) Ts cell EVs were coated onto latex beads, stained with monoclonal antibodies against CD9, CD63, and CD81 tetraspanins or against mouse antibody kappa light chains, and then analyzed by flow cytometry (n = 3, N = 2). (**C**) Ts cell EVs were separated by antigen affinity chromatography on column filled with Sepharose coated with anti-CD9 monoclonal antibodies, and the resulting fractions, i.e., flow through or eluate, were used to treat DTH effector cells prior to adoptive transfer to recipients, challenged 24 h later by id administration of the same Cas solution (5 µg per earlobe). After 24 h, ear swelling response was measured and expressed as delta ± SEM [units (U) × 10^−2^ mm] (n = 5, N = 2). (**D**) Ts cell EVs were separated by antigen affinity chromatography on column filled with Cas-coated Sepharose, and the resulting fractions, i.e., flow through or eluate, were used to treat DTH effector cells prior to adoptive transfer to recipients, challenged 24 h later by id administration of the same Cas solution (5 µg per earlobe). After 24 h, ear swelling response was measured and expressed as delta ± SEM [units (U) × 10^−2^ mm] (n = 5, N = 3). * *p* < 0.05; ** *p* < 0.01; *** *p* < 0.001; **** *p* < 0.0001.

Phenotype of suppressive cells was confirmed in positive selection according to CD8 expression. Selected, CD8+ enriched and CD8 negative cells were cultured separately for 48 h, and the pelleted EVs from ultracentrifuged culture supernatant were incubated with DTH effector cells prior to adoptive transfer. Statistically significant decrease in ear swelling was observed in challenged recipient mice had been administered with DTH effector cells incubated with EVs released by either CD8+ T cell enriched or non-selected cells from tolerized mouse lymph nodes and spleens (Figure 3A). This observation confirmed that the population of CD8+ T lymphocytes is responsible for the production of suppressive, miRNA-150-carrying EVs. By means of flow cytometry, Ts cell EVs were shown to express CD9 and CD81 tetraspanins, and CD9+ EVs eluted from anti-CD9 chromatography column suppressed DTH effector cells (Figure 3B,C). Interestingly, the flow through fraction of the anti-CD9 column increased DTH ear swelling. We assumed that this may result from the presence of Cas-specific antibody light chains non-associated with CD9+ EVs in the flow through fraction from the anti-CD9 column, as detected by flow cytometry (Figure 3B). Furthermore, Ts cell EVs expressed antibody light chains (Figure 3B), which could be responsible for their antigen specificity. To test this assumption, suppressive EVs were separated by antigen-affinity chromatography. Cas-binding EVs eluted from the Cas column, when incubated with DTH effector cells prior to adoptive transfer, significantly suppressed the elicited DTH ear swelling (Figure 3D). These results prove that suppressive EVs are Cas-specific.

### 3.4. Cas-Coupled Erythrocyte Intravenous Administration Induces Tolerance and the Resulting EVs Are Suppressive after Administration via Different Routes

**Figure 4:**
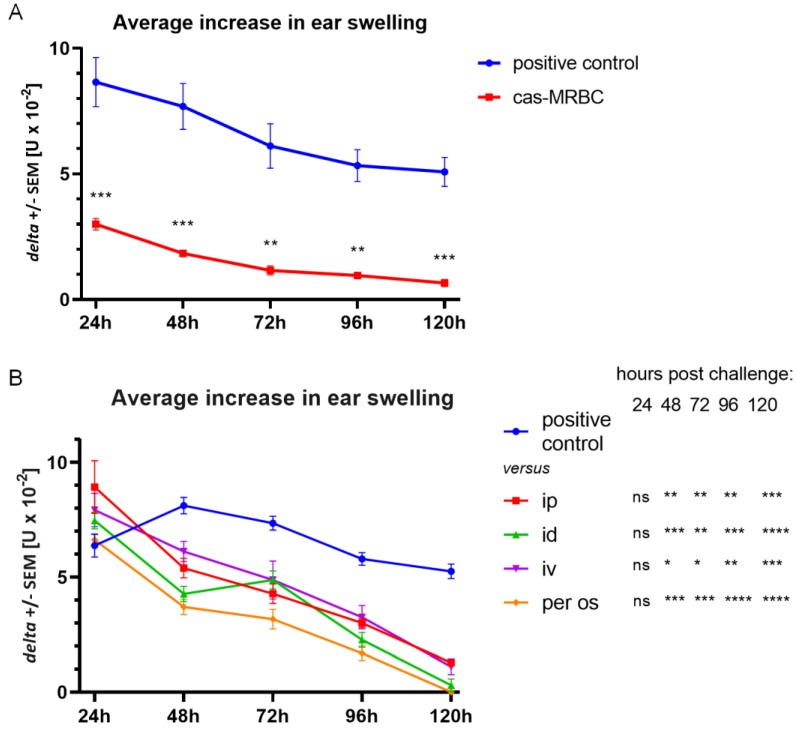
Suppression of delayed-type hypersensitivity (DTH) to soluble casein (Cas) antigen is induced by intravenous (iv) injection of Cas-coupled syngeneic red blood cells and by CD8+ suppressor T (Ts) cell-derived extracellular vesicles (EVs) administered via different routes. (**A**) Mice had been administered iv with Cas-coupled syngeneic red blood cells, which was followed by intradermal (id) immunization with a saline solution of soluble Cas antigen (100 µg per mouse) 5 days before challenging by id administration of the same Cas solution (5 µg per earlobe). Subsequent ear swelling response was measured daily up to 120 h after challenge and expressed as delta ± SEM [units (U) × 10^−2^ mm] (n = 5, N = 2). (**B**) Mice had been id immunized with a saline solution of soluble Cas antigen (100 µg per mouse) 5 days before challenging by id administration of the same Cas solution (5 µg per earlobe). After measurement of 24 h ear swelling, Ts cell EVs were administered intraperitoneally (ip), id, iv, or per os, and subsequent ear swelling response was measured daily up to 120 h after challenge and expressed as delta ± SEM [units (U) × 10^−2^ mm] (n = 5, N = 2). * *p* < 0.05; ** *p* < 0.01; *** *p* < 0.001; **** *p* < 0.0001.

Mice were tolerized with syngeneic erythrocytes conjugated with Cas antigen (Cas-MRBC), prior to intradermal immunization with Cas. Figure 4A shows the average inflammatory response after 24, 48, 72, 96, and 120 h after challenge with Cas antigen. Statistically significant decrease in ear swelling in the case of tolerized mice was then observed at every time point, which proves that intravenous tolerization with Cas-MRBC prevents the development of DTH to Cas, likely due to Ts cell EVs carrying miRNA-150.

Thus, Ts cell EVs were administered to Cas-immunized mice intravenously (iv), intradermally (id), intraperitoneally (ip), or per os, at the peak of 24 h inflammatory response. Figure 4B presents the average inflammatory response in each group, measured daily up to 120 h after challenge with Cas antigen. All administration routes induced a significant decrease in ear swelling in comparison to the positive control, with the most notable decrease observed when Ts cell EVs were administered intradermally, which is the route used for immunization of animals, and per os, which is the natural means of immunization with food protein allergens, such as Cas.

### 3.5. Ts Cell EVs Obtained from Tolerized CBA Mice Suppress the Adoptively Transferred DTH Reaction in Different Strains of Mice

**Figure 5:**
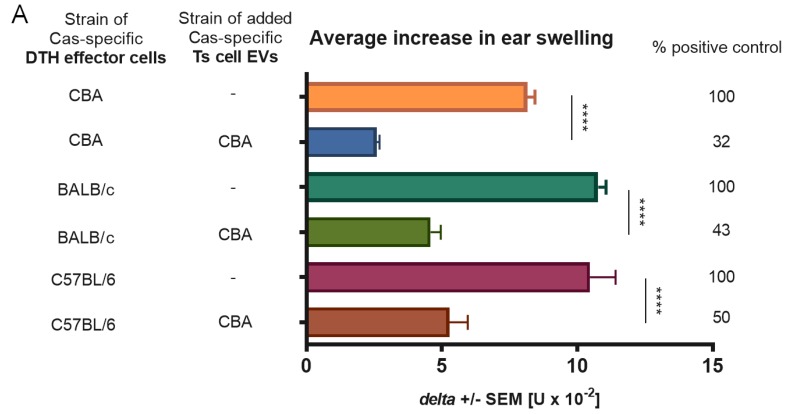

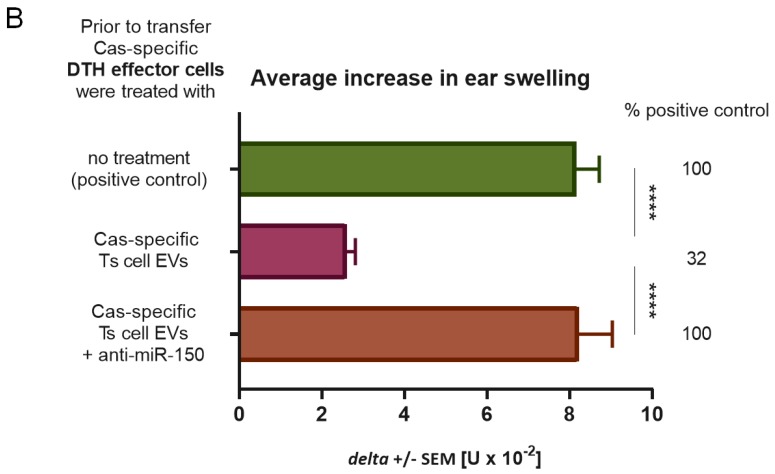
Major histocompatibility complex (MHC) restriction and dependence on miRNA-150 of suppression of delayed-type hypersensitivity (DTH) to soluble casein (Cas) antigen. (**A**) Mice of CBA, BALB/c, or C57BL/6 strains had been intradermally (id) immunized with a saline solution of soluble Cas antigen (100 µg per mouse) 5 days before harvest of lymph nodes and spleens containing effector cells, which were then treated with Cas-specific suppressor T (Ts) cell-derived extracellular vesicles (EVs) of tolerized CBA mice. Afterwards, effector cells were transferred to naive recipients of respective strain, which 24 h later were challenged by id administration of the same Cas solution (5 µg per earlobe). After 24 h, ear swelling response was measured and expressed as delta ± SEM [units (U) × 10^−2^ mm] (n = 4, N = 2). (**B**) Part of Ts cell EVs was incubated with miRNA-150 antagonist, i.e., anti-miR-150, prior to treatment of DTH effector cells, which were then adoptively transferred to recipients, challenged 24 h later by id administration of the same Cas solution (5 µg per earlobe). After 24 h, ear swelling response was measured and expressed as delta ± SEM [units (U) × 10^−2^ mm] (n = 5, N = 3). **** *p* < 0.0001.

DTH effector cells of CBA, BALB/c, or C57BL/6 mice immunized with Cas antigen were incubated with Ts cell EVs from CBA mice tolerized to Cas. Next, the effector cells were administered iv to the naive recipients of respective strain. A significant decrease in ear swelling was observed in all tested strains in groups treated with CBA Ts cell EVs, with the strongest effect detected in the case of CBA mice (Figure 5A), which suggests that EV action is rather not MHC-restricted.

### 3.6. The Blockade of miRNA-150 Activity in EVs Deprives Them of Suppressive Properties

To confirm, whether miRNA-150 is indeed the suppressive component of Ts cell EVs, they were incubated with antagonist of miRNA-150 (anti-miR-150) for 1 h before incubation with DTH effector cells from animals immunized with Cas antigen. Then, DTH effector cells were administered iv to naive mice that were ear-challenged with Cas antigen on the following day. Significant suppression of inflammatory response was observed in a group administered with DTH effector cells incubated with intact Ts cell EVs, while no significant suppression was observed in a group treated with inactivated miRNA-150 (Figure 5B). This confirms that miRNA-150 provides suppressive capacity of Ts cell EVs.

### 3.7. Cas-Specific B1 Cell-Produced EVs Gain Suppressive Activity after Incubation with miRNA-150 and Express Therapeutic Potential after Administration via Different Routes

**Figure 6:**
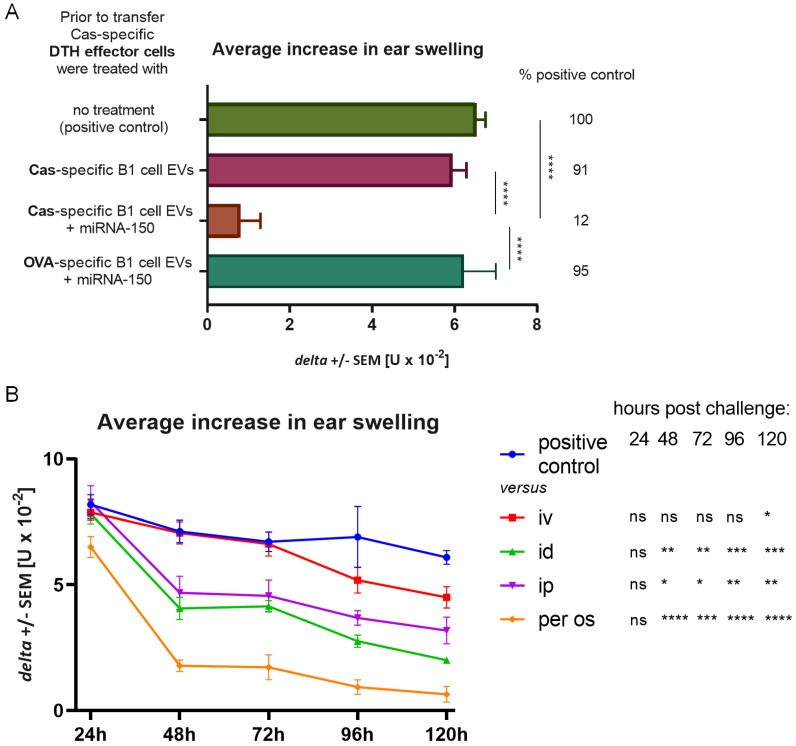
Suppression of delayed-type hypersensitivity (DTH) to soluble casein (Cas) antigen by miRNA-150-supplemented B1 cell-derived extracellular vesicles (EVs) of Cas-immunized mice. (**A**) Mice had been intradermally (id) immunized with a saline solution of soluble Cas antigen or ovalbumin (OVA, both 100 µg per mouse) 3 days before harvest of lymph nodes and spleens containing B1 cells, which were cultured for a subsequent 48 h. The resulting supernatant was filtered and ultracentrifuged to concentrate Cas-specific or OVA-specific B1 cell EVs, then supplemented with miRNA-150 prior to treatment of Cas-specific DTH effector cells, which were adoptively transferred to recipients, challenged 24 h later by id administration of the same Cas solution (5 µg per earlobe). After 24 h, ear swelling response was measured and expressed as delta ± SEM [units (U) × 10^−2^ mm] (n = 5, N = 2). (**B**) Mice had been id immunized with a saline solution of soluble Cas antigen (100 µg per mouse) 5 days before challenging by id administration of the same Cas solution (5 µg per earlobe). After measurement of 24 h ear swelling, miRNA-150-supplemented, Cas-specific B1 cell EVs were administered intraperitoneally (ip), id, intravenously (iv), or per os, and subsequent ear swelling response was measured daily up to 120 h after challenge and expressed as delta ± SEM [units (U) × 10^−2^ mm] (n = 4, N = 2). * *p* < 0.05; ** *p* < 0.01; *** *p* < 0.001; **** *p* < 0.0001.

B1 cell-derived EVs were proposed effective vehicles for transmission of regulatory miRNAs. To verify this hypothesis, we had incubated B1 cell-derived EVs from Cas-immunized mice with miRNA-150 and used them to treat Cas-specific DTH effector cells, which were next administered to naive recipients. No significant suppression of inflammatory response was observed in a group administered with B1 cell-produced EVs alone, while B1 cell EVs supplemented with miRNA-150 significantly suppressed DTH reaction (Figure 6A). On the other hand, ovalbumin-specific B1 cell EVs supplemented with miRNA-150 failed to suppress Cas-specific DTH effector cells (Figure 6A). These results prove that B1 cell EVs themselves are non-suppressive but can gain suppressive properties when supplemented with miRNA-150 and that they suppress DTH reaction afterwards in an antigen-specific manner.

To verify Cas-specific, B1 cell EVs’ therapeutic potential after supplementation with miRNA-150, they were administered to mice intravenously (iv), intradermally (id), intraperitoneally (ip), or per os at the peak of 24 h inflammatory response to Cas antigen. The most notable decrease in ear swelling response was observed in a group of mice administered with EVs per os (Figure 6B), analogously to Ts cell EVs (see Figure 4B). Statistically significant suppression was also noted in groups administered with EVs ip and id, while there were no significant differences between the positive control and the iv treated group until 120 h (Figure 6B). These results confirm that Cas-specific B1 cell EVs can gain suppressive properties when supplied with miRNA-150 and that the most effective method of their administration is the same as the natural route of immunization with Cas antigen, i.e., the oral route, which is well tolerated by patients.

## 4. Discussion

Food allergy is considered a consequence of a breakdown of oral tolerance. This process can be initiated by disturbances in antigen uptake caused by increased skin and gut permeability that lead to dissemination of the food allergen, presentation of its epitope by antigen-presenting cells (APCs), and sensitization [17]. Animals in the experiments were fed with casein-free chow in order to prevent any previous contact with the allergen and to exclude the possibility of oral tolerance development. A new mouse model of DTH to Cas was established using soluble Cas antigen administered intradermally without an adjuvant, and the inflammatory response, peaking at 24 h after challenge, was also effectively transferred from immunized donors to naive recipients, as unraveled by CD4+ T lymphocytes and macrophages as effector cells. The important role of macrophages in inflammatory reactions suggests that CD4+ T lymphocytes belong to the Th1 subpopulation. According to Lerch's and Pichler’s subclassification of cell-mediated hypersensitivity, DTH to Cas can be classified as a IVa allergic reaction [6]. 

EVs and their ability to transport bioactive molecules, such as miRNAs, to acceptor cells are a new, promising tool that may be used in diagnosis and therapy for a variety of human diseases [18,19]. Bai et al. reported that miRNA-150 may inhibit the proliferation and promote the cell cycle arrest in thyroid cancer cells [20]. Exogenous miRNA-150 was also described to inhibit proliferation and metastasis and to enhance cell apoptosis in human osteoblasts [21]. Chen et al. investigated the therapeutic effect of mesenchymal stem cell-derived EVs carrying miRNA-150-5p in potential therapy of rheumatoid arthritis. Injection of miRNA-150-loaded EVs reduced joint destruction by inhibition of hyperplasia and angiogenesis in a mouse model of collagen-induced arthritis [22]. miRNA-150 was proved to be a regulatory factor carried by Ts cell-derived exosomes [11]. Suppressive capacity of Ts cell EVs was confirmed by numerous independent groups in a mouse model of contact sensitivity to haptens [13]. We managed to separate Ts cell EVs from cell culture supernatant from animals tolerized to food protein, such as Cas, and to prove its ability to suppress inflammatory reaction in DTH to Cas antigen both in adoptive transfer and in active immunization. Cas-specific Ts cell EVs treated with miRNA-150 antagonist prior to incubation with Cas DTH effector cells proved to be inactive. Additionally, B1 cell-derived EVs did not suppress DTH reaction to Cas antigen, unless they were supplemented with miRNA-150. These results confirmed that Ts cell EVs, produced by animals tolerized to Cas antigen, owe their suppressive activity to miRNA-150 that is carried by EVs derived from CD8+ Ts cells and not B1 cells. 

We confirmed that suppressive EVs in DTH to Cas, like in CS to haptens, were a product of CD8+ Ts lymphocytes by means of positive selection. Furthermore, antigen-specificity of Ts cell EVs was tested in antigen-affinity chromatography and the eluate from the column proved to be strongly suppressive in contrast to flow through. Bryniarski et al. tested antigen-specificity of EVs in CS to haptens in both antigen-affinity chromatography and in a crisscross experiment, where trinitrophenol-specific Ts cell EVs failed to suppress inflammatory reaction to oxazolone. The antigen-specificity of EVs was then demonstrated by means of flow cytometry to be the result of IgM antibody light chain (LC) coating [11]. Those LCs are produced by B1 lymphocytes activated by immunization with an antigen, which follows the tolerization with antigen-coupled syngeneic red blood cells [15]. Current results demonstrated the presence of LC on Cas-specific Ts cell EVs, which additionally confirms their antigen-specificity. Furthermore, miRNA-150-supplemented B1 cell-derived EVs from mice immunized with ovalbumin failed to suppress Cas-induced DTH, which brought more evidence for the LC-mediated specificity of EV action. IgM antibodies produced by B1 lymphocytes are usually characterized by low specificity to antigen as their secretion does not require signals from helper T cells. Lately, it was presented that a special subset of B1a cells generates high antigen-affinity IgM antibodies and free LCs as a consequence of immunoglobulin V-region mutations induced by activation-induced cytidine deaminase. B1a lymphocytes are suggested to initiate early responses in immune resistance to pneumococcal pneumonia, CS, and DTH [23,24], which was herein indirectly confirmed by the enhancement of DTH reaction caused by the flow through fraction of the anti-CD9 column, demonstrated to contain Cas-binding LC. Recent findings confirm that B1 cell-derived LCs provide the specificity of EVs in CS to haptens and enable execution of their suppressive function, as the Ts cell EVs of B-cell-deficient or immunoglobulin-deficient mice are non-suppressive [25]. Previously, it was demonstrated that injection of haptenized MRBC before contact sensitization with oxazolone and elicitation of CS ear swelling response leads to suppression of inflammatory response in wild-type mice [25]. Here, we confirmed that double injection of cas-MRBC prior to immunization with the Cas antigen protected animals from developing of DTH reaction to Cas. The results suggest that animals tolerized with Cas antigen actively produce Ts cell EVs that prevent elicitation of inflammatory response up to 120 h. 

Ts cell EVs modulate inflammatory response indirectly. Transfer of effector cells incubated with peritoneal macrophages treated previously with Ts cell EVs inhibited reaction in CS effector cell recipients [26]. Lately, it was confirmed that EVs containing miRNA-150 act as mediators in communication between effector T cells and APC, since mice depleted of macrophages cannot be effectively tolerized [27]. APCs express major histocompatibility complex class II (MHC II) on their surface that was reported to be also expressed on their EVs, which enables targeting of CD4+ T cells [28]. Here, we investigated whether the suppressive effect of Ts cell EVs depends on MHC II by treatment of DTH effector cells from CBA, BALB/c, and C57BL/6 mice immunized with Cas with EVs collected from CBA mice tolerized to Cas antigen. In our experiment, CBA Cas-specific Ts cell EVs were effective in each strain. This suggests that T cell-derived EVs, in contrast to T cells themselves, cannot check MHC homology, which implies that those EVs, like miRNAs, may mediate a highly conserved mode of communication across strains. 

We tested four different routes of Ts cell EV administration and all of them effectively suppressed inflammatory response in active immunization. Interestingly, the most effective routes of EV administration, i.e., id and per os, were also the routes of immunization with the antigen. In our experiments, animals were immunized intradermally and the natural route of immunization with food protein allergens would be through the gastrointestinal tract. Furthermore, the oral route is also the natural means of food tolerance induction. Intestinal epithelial cell-derived EVs, carrying αvβ6 integrin and food antigens, were reported to stimulate tolerance in dendritic cells (DCs) and promote regulatory T cell development in a model of oral tolerance induction [29]. Previously, DCs were described to directly sample the content of intestine lumen through the trans-epithelial dendrites [17,30]. Food antigens could also be internalized in Peyer’s patches and captured by APCs, which then migrate to lymph nodes and activate effector or regulatory T cells [31]. These mechanisms of oral tolerance create a suitable opportunity for Ts cell EVs to reach APCs and transfer the suppressive information. In a following experiment, we tested the same routes of administration of miRNA-150-supplemented B1 cell EVs, and the oral administration again happened to be the most effective. Statistically significant differences were also observed when EVs were administered ip and id, which is possibly a result of particularly easy access to resident macrophages and DCs in peritoneal cavity and skin [32,33]. Intravenous injection of the miRNA-150-supplemented B1 cell EVs to Cas-immunized mice was not effective until 120 h in contrast to previous experiments with Ts cell EVs, where all routes suppressed inflammatory reaction within 24 h of administration. Our results may indicate that miRNA-150 is not internalized by B1 cell EVs but adheres to their surface instead, where it is susceptible to enzymatic hydrolysis. In contrast, when miRNA-150 is originally sorted to EVs in MVB of Ts cells, it is protected from ribonucleases activity in serum. Thus, miRNA-150-supplemented B1 cell EVs require more time to access sufficient amount of APCs to deliver the suppressive information.

It is worth noting that EVs are resistant to harsh conditions, including very low pH and activity of digestive enzymes, which enables the protection of contained RNA cargo, as reported in the case of the transmission of dietary miRNAs associated with EVs via intestines [34,35]. This was true also for maternal milk exosome RNA cargo transferring epigenetic information to neonates after intestinal absorption [36], likely via epithelial cell endocytosis [37,38]. Interestingly, EV-associated miRNAs absorbed via intestinal barrier have been proposed to regulate immunity [39], and our current results seem to confirm this assumption. 

## 5. Conclusions

We standardized a method to produce immunogenic, soluble Cas antigen activating DTH when administered intradermally without an adjuvant. Further, we developed a new model of DTH to a milk protein allergen and identified the phenotype of effector cells responsible for the inflammatory reaction as CD4+ Th1 lymphocytes and macrophages. The possibility of antigen-specific suppression of cell-mediated reaction in allergic response to food protein, such as Cas, was also confirmed. Suppressive EVs obtained from cell supernatants of mice tolerized to Cas were a product of CD8+ Ts lymphocytes. miRNA-150 was described as the suppressive compound of Cas-specific Ts cell EVs. Animals tolerized to Cas prior to immunization actively produced Ts cell EVs that protected them from developing DTH response to Cas. The oral route of administration was the most effective for treatment with either Ts cell EVs or miRNA-150-supplemented B1 EVs, as it is the natural route of oral tolerance development. Cas-specific Ts cell EVs derived from CBA mice suppressed inflammatory reactions in different strains of mice immunized to Cas antigen, which suggests that the intercellular communication via suppressive EVs is conserved among strains.
